# Effects of fentanyl administration in mechanically ventilated patients in the intensive care unit: a systematic review and meta-analysis

**DOI:** 10.1186/s12871-022-01871-7

**Published:** 2022-10-21

**Authors:** Yoshitaka Aoki, Hiromi Kato, Naoyuki Fujimura, Yuji Suzuki, Masaaki Sakuraya, Matsuyuki Doi

**Affiliations:** 1 Department of Anesthesiology and Intensive Care Medicine, Hamamatsu University School of Medicine, 1-20-1 Handayama, Higashi-ku, Hamamatsu, Shizuoka 431-3192 Japan; 2 Department of Anesthesiology, St. Mary’s Hospital, Kurume, Fukuoka Japan; 3 Department of Emergency and Intensive Care Medicine, JA Hiroshima Hospital, Hiroshima, Japan

**Keywords:** Fentanyl, Opioid, Mechanical ventilation, Remifentanil, Morphine

## Abstract

**Background:**

Fentanyl is selected to manage pain in critical care patients on mechanical ventilation in the intensive care unit (ICU). However, the usefulness of fentanyl compared with other opioids is unknown. This study examined the evidence for using fentanyl to improve the clinical outcomes of ICU patients, using the Grading of Recommendations Assessment, Development, and Evaluation (GRADE) system.

**Methods:**

We searched the MEDLINE, Cochrane Central Register of Controlled Trials, and Igaku Chuo Zasshi databases in June 2021. Two independent assessors reviewed studies to identify randomized, controlled trials (RCTs) that compared the intravenous administration of fentanyl and other opioids in mechanically ventilated patients in the ICU. The study quality was assessed using the GRADE system and Cochrane methodology. The primary outcome was mortality. The secondary outcomes were the duration of mechanical ventilation, duration of the ICU stay, incidence of severe adverse events, and incidence of delirium. We integrated outcome data using a random-effects model and showed absolute values and certainty of evidence in the GRADE evidence profile.

**Results:**

Seven RCTs met the study inclusion criteria with 534 patients (251 were treated with fentanyl and 283 with other opioids, including 242 with remifentanil and 41 with morphine). Among 191 participants from 2 RCTs, fentanyl was not associated with mortality (risk ratio [RR], 0.79; 95% confidence interval [CI], 0.24 to 2.60; low-quality evidence). Regarding the secondary outcomes, fentanyl did not shorten the duration of mechanical ventilation (mean difference, 0.49 h; 95% CI, − 0.90 to 1.88; moderate-quality evidence) or the duration of the ICU stay (mean difference, 7.04 h; 95% CI, − 3.27 to 17.35; moderate-quality evidence) compared with other opioids. Fentanyl did not increase the incidence of severe adverse events (RR, 0.98; 95% CI, 0.50 to 1.90; low-quality evidence) or delirium (RR, 1.27; 95% CI, 0.79 to 2.04; low-quality evidence).

**Conclusions:**

Although fentanyl is a frequently administered opioid in the ICU, patients’ outcomes are not different between fentanyl use and use of other opioids. However, the GRADE evaluation provides little certainty to support the results of this systematic review. Therefore, further large RCTs are required to confirm our conclusions.

**Trial registration:**

PROSPERO, CRD42019130648 (https://www.crd.york.ac.uk/prospero/display_record.php?RecordID=130648).

**Supplementary Information:**

The online version contains supplementary material available at 10.1186/s12871-022-01871-7.

## Background

Pain management is an important issue for critically ill adults in the intensive care unit (ICU), and inadequate pain management may lead to posttraumatic stress disorder [1] and post-intensive care syndrome [2, 3]. The Pain, Agitation/Sedation, Delirium, Immobility, and Sleep disruption guidelines recommend a continuous infusion of opioids for procedural pain management in critically ill adults [4]. However, the same opioids (i.e., fentanyl, hydromorphone, morphine, and remifentanil) have consistently been recommended since 2013. There has been no systematic review on the use of opioids for pain management in critically ill adults [5]. Therefore, the appropriate analgesic drugs to use remain controversial.

Fentanyl is a potent, selective 4-anilidopiperidine μ-opioid analgesic used for analgesic management in mechanically ventilated patients in the ICU [6, 7]. Among the other available opioids, morphine has common adverse effects, such as histamine release, pruritus, and accumulation of morphine-6-glucuronide, in patients with renal impairment [8]. Additionally, neither alfentanil nor sufentanil is licensed for use in ICU patients in many countries [8]. Hydromorphone also appears to be used for mechanically ventilated patients in the ICU [9], but few studies have reported its usefulness. Moreover, remifentanil has been reported to slightly shorten the duration of mechanical ventilation, the time to extubation after ceasing sedation, and the ICU stay [10]. However, this drug is not commonly administered in the ICU because of its side effects of acute tolerance and hyperalgesia [11]. Therefore, fentanyl is a relatively common choice of opioid used in multinational, randomized, controlled trials (RCTs) [12] and especially in Japanese ICUs [13]. However, no systematic review of fentanyl intervention and no evidence of the benefits of fentanyl have been provided yet.

We hypothesized that fentanyl is associated with better outcomes than other opioids in mechanically ventilated adults in the ICU. Therefore, this meta-analysis evaluated the effects of fentanyl by analyzing the results of previous RCTs that compared fentanyl with other opioids and integrated the outcomes on the basis of the Grading of Recommendations Assessment, Development, and Evaluation (GRADE) system.

## Materials and methods

This systematic review and meta-analysis were conducted according to the Preferred Reporting Items for Systematic Reviews and Meta-Analyses (PRISMA) statement [14] and the GRADE system [15]. The study was exempt from ethics review and did not require written informed consent. The study was registered with the International Prospective Register of Systematic Reviews (PROSPERO, number CRD42019130648). This systematic review was conducted as part of the revision of the Japanese Clinical Practice Guidelines for Management of Sepsis and Septic Shock 2020 (J-SSCG 2020). The present study was initially conducted in 2019 for the J-SSCG 2020, but we conducted a literature search again in 2021 to avoid double submissions.

### Search strategy

We electronically searched the MEDLINE (PubMed), Cochrane Central Register of Controlled Trials (CENTRAL), and Igaku Chuo Zasshi (largest database of Japanese medical journals) databases in June 2021. We used search strategies according to the Cochrane Handbook for Systematic Reviews of Interventions [16]. Additionally, we searched for ongoing trials in ClinicalTrials.gov and the World Health Organization International Clinical Trials Platform Search Portal. The following keywords were used for the search strategy: “acute lung injury,” “critical care,” “multiple organ failure,” “sepsis,” “ventilation, mechanical,” “fentanyl,” “opioid,” and “randomized controlled trial” (Additional file 1). We also carried out a manual search of the reference lists of the identified studies. We limited our search to articles published in Japanese or English.

### Study inclusion criteria

We selected RCTs that compared the use of fentanyl versus other opioids in mechanically ventilated adults in the ICU. We included mechanically ventilated adults in the ICU who were intravenously administered analgesia with fentanyl or other opioids. Exclusion criteria were drugs administered only before ICU admission (e.g., during surgery), non-intravenous administration (e.g., intrathecal administration), patients younger than 18 years, crossover design, and articles in languages other than Japanese and English. The outcomes were selected by an independent committee based on a preliminary assessment of the importance of the outcomes, without members of the systematic review, following the GRADE clinical guideline development process. The primary outcome was mortality, which was defined as the longest period over which mortality was assessed in each article. The secondary outcomes were the duration of mechanical ventilation, duration of the ICU stay, incidence of severe adverse events, and incidence of delirium.

### Data collection

The titles and abstracts of all of the extracted studies were screened independently and assessed by two authors (H.K., H.F.) according to our inclusion and exclusion criteria. Data elements were extracted to confirm the study eligibility, study design, patients’ demographics, performed interventions, outcomes of interest, statistical methods, and study results. All inconsistencies during data extraction were resolved by a third author (Y.A.) to reach a consensus.

### Assessment of risk of bias

The qualities of the included studies were assessed independently by two authors (H.K., H.F.) according to the Cochrane methodology [16]. In case of disagreement, the final decision was made by a third author (Y.A.). The extent of potential bias in the included studies was assessed using the Cochrane “risk of bias” tool in RevMan 5.4 (Copenhagen: The Nordic Cochrane Centre, The Cochrane Collaboration, 2014). We considered the following domains: random sequence generation, allocation concealment, blinding of participants and personnel, blinding of outcome assessment, incomplete outcome data, selective reporting, and other biases. We evaluated each methodological quality item as “yes,” “no,” or “unclear” (owing to no or less clear reporting) for each eligible study and created a “risk of bias” summary. We then evaluated the overall validity of each study as a low, intermediate, or high risk of bias.

### Data synthesis and statistical analysis

We carried out the statistical analysis using RevMan 5.4. We used a random-effects model for combined data where the assumption that the studies estimated the same underlying treatment effect was reasonable. Forest plots were constructed to display the results of the individual studies and pooled estimates of effect. The pooled dichotomous outcomes are expressed as the risk ratio (RR) and 95% confidence interval (CI). A pooled estimate of the treatment effect was calculated as the mean difference (MD) and 95% CI for continuous variables. Data provided as the median and interquartile range (or range) were converted to the mean and standard deviation, where appropriate, to calculate pooled RRs and MDs [17]. The forest plot displays other types of opioids (remifentanil and morphine) in the control group as subgroups. In a sensitivity analysis, we excluded studies in which the intervention and comparison groups differed in co-interventions (e.g., sedatives other than opioids). Heterogeneity across studies was tested using the I^2^ statistic. We investigated reporting bias (publication bias) using a funnel plot and visually assessed the funnel plot asymmetry. Finally, we summarized the results in evidence profiles using the GRADE approach. An independent peer review committee reviewed the GRADE evidence profile, and a third party checked each measurement for validity.

## Results

### Search results

**Fig. 1:**
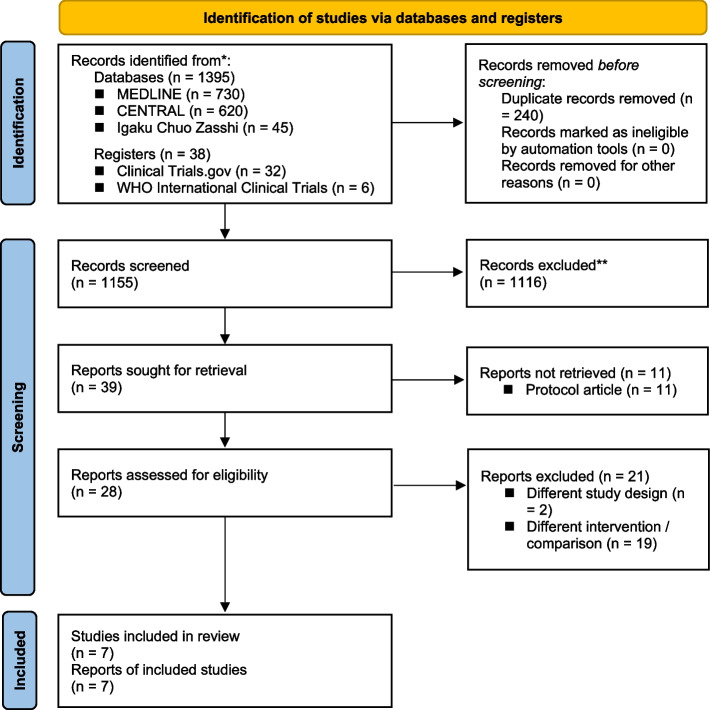
PRISMA (Preferred Reporting Items for Systematic Reviews and Meta-Analyses) flow diagram. CENTRAL, Cochrane Central Register of Controlled Trials; WHO, World Health Organization; MV, mechanical ventilation; ICU, intensive care unit

On 22 June 2021, we identified 730, 620, and 45 studies from searches of MEDLINE, CENTRAL, and Igaku Chuo Zasshi, respectively. In addition, as with other sources, we identified 32 studies from ClinicalTrials.gov and 6 studies from World Health Organization International Clinical Trials (Additional file 1). Manual searching of the reference lists did not show any additional publications. Our electronic database search identified 1155 studies, of which 1116 were excluded after applying the inclusion and exclusion criteria. After reviewing the full-text articles, a further 21 studies were excluded. The studies that appeared to meet the inclusion criteria, but were excluded, are summarized in Additional file 2, along with the reasons for exclusion. Therefore, seven studies remained for the systematic review (Fig. 1).

### Design and characteristics of included studies

The seven included studies are shown in Table 1 [18–24]. A total of 534 participants were included, of whom 251 received fentanyl (intervention group) and 283 received other opioids (242 had remifentanil and 41 had morphine; control group). Three studies had two control groups in contrast to the one fentanyl intervention group [19, 20, 23]. Therefore, we used the larger sample size of the two control groups in this study. Because this systematic review showed significant differences in the characteristics of the patients and the indications for sedatives and opioids, details are provided in Additional file 3. Outcomes included in the meta-analysis are listed in Additional file 4 as they appear in the respective literature. In the sensitivity analysis, we excluded two studies in which co-intervention of sedatives differed between the intervention and comparison groups from the analysis [19, 22].

**Table 1:** Characteristics of included studies

Authors	Country	Setting	Study period	Total number of patients randomized	Patients’ conditions	No. of study arms	Interventions	Comparisons	Outcomes
Cevik et al. [18]	Turkey	Single-center, open-label	September 2007 to May 2008	34	Patients requiring MV and sedation in the ICU	2	Fentanyl-midazolam regimen	Remifentanil-midazolam regimen	• Hemodynamic parameters • Analgesics and sedative dosage • Mean MV time^a^ • Mean time to ICU discharge^a^ • Organ dysfunction • Adverse effect^a^
Karabinis et al. [19]	Six countries in Europe (4 hospitals in Greece, 4 in Spain, 3 in Belgium, 3 in The Netherlands, 2 in Germany, 1 in Austria)	Multi-center, open-label	Not stated (published in 2004)	161	Acute, severe neurological insult or injury, or patients who had undergone elective or emergency neurosurgery	3	Hypnotic-based treatment (fentanyl)	Remifentanil-based treatment	• Median time on MV^a^ • Extubation process • Extubation to ICU discharge^a^ • Serious adverse events^a^ • Drug-related serious adverse events • Mortality^a^
Liu et al. [20]	China	Single-center, double-blind	September 2014 to January 2015	105	MV anticipated for > 24 h	3	Fentanyl	Remifentanil	• 28-day mortality^a^ • Duration of MV^a^ • Length of the ICU stay^a^ • Delirium^a^ • Duration of delirium • Weaning time
Muellejans et al. [21]	Five countries, 21 centers (4 centers in Belgium, 8 in Germany, 1 in the Netherlands, 4 in Spain, 4 in the UK)	Multi-center, double-blind	Not stated (published in 2004)	152	MV for a further 12 to 72 h	2	Fentanyl	Remifentanil	• Extubation process – extubation^a^ • Extubation – ICU discharge • Drug start—ICU discharge^a^ • Adverse events • Drug-related adverse events • MAP < 50 mmHg • HR < 50 beats/min • Severe adverse events^a^
Muellejans et al. [22]	Germany	Single-center, open-label	Not stated (published in 2006)	80	MV for > 12 h	2	Hypnotic-based sedation with midazolam/fentanyl	Remifentanil-based analgesia and sedation with propofol	• Any adverse event • Any serious adverse event^a^ • Delirium^a^ • Time from arrival in the ICU to extubation^a^ • Time from arrival in the ICU to eligible ICU discharge^a^ • Overall costs
Oliver et al. [23]	United States	Single-center, observer-blind	Not stated (published in 2011)	113	Scheduled elective cardiac surgery	3	Fentanyl and propofol	Morphine and propofol	• Time to extubation^a^ • First response time • Length of the ICU stay^a^ • Length of the hospital stay • ICU direct medical costs
Spies et al. [24]	Germany	Two-center, double-blind	December 2005 to June 2008	60	MV for > 24 h	2	Fentanyl	Remifentanil	• Proportion of patients who obtained the target analgesia score • Duration of MV^a^ • Duration of the ICU stay^a^ • Duration of the hospital stay • Delirium^a^

*MV* Mechanical ventilation, *ICU* Intensive care unit, *CABG* Coronary artery bypass grafting, *PCSU* Postcardiac surgical unit, *MAP* Mean arterial pressure, *HR* Heart rate

^a^ Outcomes included in the meta-analysis in this study

### Effects of interventions

We appraised the certainty of the evidence using the GRADE approach and summarized the results in evidence profiles (Table 2). We found no clinically significant differences in the relative significance and no clinical differences in absolute values for all outcomes. We judged the certainty of evidence for mortality as low, the duration of ventilation and the ICU stay as moderate, and severe adverse events and delirium as low. The reasons for downgrading the certainty of evidence are explained in the footnote to Table 2.

**Table 2:** Evidence profiles

Certainty assessment							No. of patients		Effect		Certainty	Importance
**No. of studies**	**Study design**	**Risk of bias**	**Inconsistency**	**Indirectness**	**Imprecision**	**Other considerations**	**Fentanyl**	**Other Opioids**	**Relative** **(95% CI)**	**Absolute** **(95% CI)**		
**Mortality**												
2	Randomized trials	Not serious	Not serious	Not serious	Very serious ^a^	None	4/72 (5.6%)	8/119 (6.7%)	**RR 0.79** (0.24 to 2.60)	**14 fewer per 1000** (from 51 fewer to 108 more)	⨁⨁◯◯ LOW	CRITICAL
**Duration of mechanical ventilation**												
7	Randomized trials	Not serious	Serious ^b^	Not serious	Not serious	None	251	283	-	MD **0.49 higher** (0.9 lower to 1.88 higher)	⨁⨁⨁◯ MODERATE	CRITICAL
**Duration of the ICU stay**												
7	Randomized trials	Not serious	Serious ^c^	Not serious	Not serious	None	251	283	-	MD **7.04 higher** (3.27 lower to 17.35 higher)	⨁⨁⨁◯ MODERATE	CRITICAL
**Severe adverse events**												
4	Randomized trials	Not serious	Not serious	Not serious	Very serious ^a^	None	13/173 (7.5%)	15/255 (5.9%)	**RR 0.98** (0.50 to 1.90)	**1 fewer per 1000** (from 29 fewer to 53 more)	⨁⨁◯◯ LOW	CRITICAL
**Delirium**												
3	Randomized trials	Serious ^d^	Not serious	Not serious	Serious ^e^	None	30/106 (28.3%)	23/103 (22.3%)	**RR 1.27** (0.79 to 2.04)	**60 more per 1000** (from 47 fewer to 232 more)	⨁⨁◯◯ LOW	CRITICAL

*CI* Confidence interval, *RR* Risk ratio, *MD* Mean difference, *ICU* Intensive care unit

^a^ The assessment was downgraded by two levels because it did not meet the optimal information size and the 95% CI spanned 0.75 to 1.25, which was the threshold for judgment

^b^ The grade was downgraded by one level because I^2^ for heterogeneity was 93%

^c^ The grade was downgraded by one level because I^2^ for heterogeneity was 93%

^d^ Muellejans et al. (28.9% weight of all results) used different sedatives in the intervention and control groups, and Spies et al.’s study (29.2% weight of all results) was terminated early and downgraded by one level owing to the high risk of bias

^e^ The assessment was downgraded by one level because it did not meet the optimal information size and the 95% CI spanned 1.0 to 1.25, which was the threshold for judgment

### Outcome

A forest plot of all outcomes is shown in Additional file 5. Two studies were included [19, 20], and the only opioid used in the control group was remifentanil. Four (4/72) patients in the fentanyl group and eight (8/119) in the other opioids group died, which indicated that fentanyl was not associated with decreased mortality (RR, 0.79; 95% CI, 024 to 2.60). Among the secondary outcomes, the MD for the duration of mechanical ventilation was 0.49 h (95% CI, − 0.90 to 1.88) in 7 RCTs comprising 534 patients. Additionally, the MD for the duration of the ICU stay was 7.04 h (95% CI, − 3.27 to 17.35) in 7 RCTs comprising 534 patients. With regard to the duration of mechanical ventilation and the ICU stay, opioids in the control group were mixed with remifentanil and morphine, but there was no difference in the MD results following a subgroup analysis according to other opioid types. The RR of severe adverse events in 4 RCTs comprising 428 patients was 0.98 (95% CI, 0.50 to 1.90), and the RR of delirium was 1.27 (95% CI, 0.79 to 2.04) in 3 RCTs comprising 209 patients. Additional file 6 shows the sensitivity analysis results, which were similar to those for the primary analysis.

### Risk of bias in included studies

**Fig. 2:**
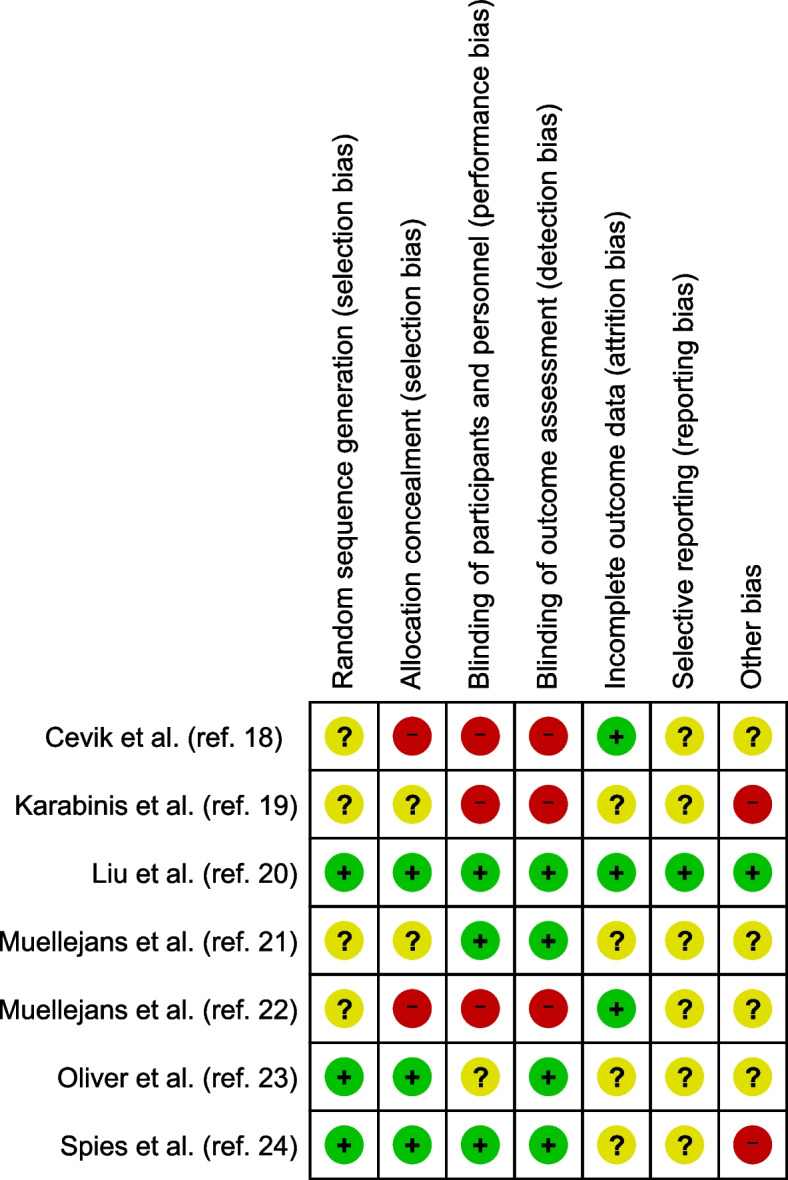
Risk of bias summary. Green circles indicate a low risk of bias, yellow circles indicate an unclear risk, and red circles indicate a high risk

The risk of bias assessments for the included studies are summarized in Fig. 2. All domains were a “low risk of bias” in one study [20], but the remaining studies lacked clarity in several domains.

### Assessment of reporting bias

We were unable to create a forest plot because fewer than 10 studies were included.

## Discussion

This meta-analysis aimed to investigate the effects of fentanyl administration in mechanically ventilated patients in the ICU using the GRADE system. We found that fentanyl was not associated with a decrease in mortality, duration of mechanical ventilation, duration of the ICU stay, incidence of serious adverse events, or incidence of delirium compared with other opioids. The results were also robust in the sensitivity analysis. However, the most common opioid that was compared with fentanyl was remifentanil. Additionally, the GRADE certainty rating of the results was rated as moderate to low, suggesting that the evidence was inadequate, and the results could thus not be confirmed.

To the best of our knowledge, this is the first meta-analysis to examine the effectiveness of fentanyl administration for mechanically ventilated patients in the ICU. Fentanyl is an opioid used for pain relief in mechanically ventilated ICU patients worldwide [6, 7, 12]. Fentanyl is a synthetic opioid with a good profile, but undergoes hepatic metabolism, and thus accumulates in tissues following continuous infusion, resulting in prolonged drug effects [25]. The Pain, Agitation/Sedation, Delirium, Immobility, and Sleep disruption guidelines state that all opioids are equally effective [4]. However, recent reports have indicated differences in postoperative complications among opioids used for postoperative patient-controlled analgesia [26], differences in the rates of maintenance of light sedation in the ICU [27], and differences in opioid-withdrawal syndrome in the ICU [28]. Another important finding of this study is that remifentanil was usually compared with fentanyl in the ICU, and no RCTs compared fentanyl with alfentanil, sufentanil, or hydromorphone. Therefore, the optimal opioid for use in the ICU is controversial, and further research is required.

The current analysis showed no difference in mortality between fentanyl and other opioids. In contrast, however, a recent propensity score-matched cohort study that compared fentanyl and morphine as analgesics in patients with acute respiratory distress syndrome reported a lower mortality with fentanyl [29]. The present meta-analysis showed no significant difference in the duration of mechanical ventilation or the ICU stay between patients treated with fentanyl or other opioids. These results remained similar even after subgroup and sensitivity analyses, which showed no difference between fentanyl and remifentanil. This result is in contrast to a systematic review that showed a slight reduction in the duration of mechanical ventilation and the ICU stay between patients treated with remifentanil and those treated with other opioids [10]. There were no differences in severe complications between the fentanyl and other opioid groups in this study. Although some studies have reported adverse effects of fentanyl in patients in the community [30], there have been few reports of severe complications in the ICU [19, 21, 31]. There was no difference in the rate of delirium between the fentanyl and other opioid groups in our analysis. However, in a relatively small cross-sectional study, fentanyl treatment did not increase the incidence of delirium, but was associated with subsyndromal delirium (defined as a score of between 1 and 3 on the Intensive Care Delirium Screening Checklist) [32]. This inconsistency among studies may be due to the small number of RCTs that compared fentanyl with other opioids, the small sample size, and different patients’ backgrounds and interventions/controls.

Following this systematic review, the independent panel committee of the J-SSCG 2020 decided against issuing a recommendation addressing this clinical question. Fentanyl is currently the only opioid that can be legally administered in Japanese ICUs. The panel committee decided that the results of this review, which did not support the use of fentanyl, might confuse the clinical practice of sepsis management. Because inadequate analgesia is associated with a worse prognosis [1–3], the choice of opioids for patients in the ICU remains an important issue for future consideration.

This study had several limitations. First, this systematic review was conducted as part of the Japanese sepsis guidelines, and experts outside the systematic review team determined the outcomes. Therefore, although opioids in the ICU are sometimes discussed in terms of cost, it was not chosen as an outcome for this study because the cost was judged not to be a critical outcome in the ventilatory management of critically ill patients. Second, the number of included studies was relatively small and the sample size was small. Therefore, a subgroup analysis and sensitivity analysis were conducted, but the robustness of this meta-analysis may be debatable. Third, the dosing protocols of the opioids administered in each study varied, which may have affected the results of this analysis. Therefore, we carefully reviewed the dosing protocols for sedatives and opioids (Additional file 3). The effects of opioids may also vary depending on the patient’s liver and kidney function. However, protocols that adjust drug doses using patients’ assessment scores, such as the Richmond Agitation Sedation Scale, may reduce the differences in drug types. Fourth, the comparison of fentanyl with other opioids was in the short term within hospitalization or 28 days, and no long-term (e.g., > 6 months) comparison has been performed. Opioids may be associated with medium- and long-term outcomes, such as post-intensive care syndrome and chronic pain, but no studies have evaluated these outcomes. Fifth, the present study was initially conducted in 2019 for the J-SSCG 2020, but was re-performed with a more recent literature search in 2021. Therefore, there are slight differences from the PROSPERO registration. An example of these differences is that alfentanil and sufentanil, which are unavailable in Japan, were excluded from the PROSPERO registration in 2019. However, we did not exclude alfentanil and sufentanil from the second search in 2021. A second literature search may have increased the generalizability of the study results. Finally, assessing the certainty of the evidence is subjective, and some researchers may not agree with our assessment. However, the present study was conducted following the methodology of the J-SSCG 2020, which included independent validation of search formulas, an external audit of systematic review work, and a peer review of the GRADE evidence profile by an independent committee. Despite these limitations, we believe that our results, which were obtained using the appropriate procedures and evaluated with the GRADE approach, have important implications for clinicians.

In conclusion, the present study used the GRADE system to examine the effect of fentanyl on mechanically ventilated patients in the ICU. We did not find any significant difference in patients’ outcomes between the use of fentanyl and other opioids. However, remifentanil was usually the only opioid compared with fentanyl, and the GRADE certainty ratings were generally low, indicating a lack of evidence in this area.

## Supplementary Information

**Additional file 1.** Search strategies.

**Additional file 2.** Relevant excluded studies and reasons for exclusion.

**Additional file 3.** Characteristics of included patients, and details of sedatives and opioids.

**Additional file 4.** Outcomes of included studies.

**Additional file 5.** Forest plot of all outcomes.

**Additional file 6.** Forest plot of all outcomes in the sensitivity analysis.

## Data Availability

The datasets used and/or analyzed during the current study are available from the corresponding author on reasonable request.

## Abbreviations

ICU: Intensive care unit
GRADE: Grading of Recommendations Assessment, Development, and Evaluation
RCTs: Randomized, controlled trials
PRISMA: Preferred Reporting Items for Systematic Reviews and Meta-Analyses
J-SSCG2020: Japanese Clinical Practice Guidelines for Management of Sepsis and Septic Shock 2020
CENTRAL: Cochrane Central Register of Controlled Trials
