# Supplementary material for: Effects of fentanyl administration in mechanically ventilated patients in the intensive care unit: a systematic review and meta-analysis
Source: BMC Anesthesiol. 2022 Oct 21;22:323. doi: 10.1186/s12871-022-01871-7 (PMC9585711; doi:10.1186/s12871-022-01871-7)
Supplement: Supplementary file 1 — Additional file 1. Search strategies. [file 12871_2022_1871_MOESM1_ESM.pdf]

Additional file 1. Search strategies.

PubMed search strategy (search date: 22 June 2021)

#1	(“acute lung injury”[mesh] OR “burn units”[mesh] OR “critical care”[mesh] OR “critical illness”[mesh] OR “intensive care units”[mesh] OR “intubation”[mesh] OR “intubation, intratracheal”[mesh] OR “multiple organ failure”[mesh] OR “respiratory care units”[mesh] OR “respiration, artificial”[mesh] OR “respiratory distress syndrome, adult”[mesh] OR “sepsis”[mesh] OR “shock”[mesh] OR “shock, septic”[mesh] OR “ventilators, mechanical”[mesh] OR “bacteremia”[mesh] OR “endotoxemia”[mesh] OR “hemorrhagic septicemia”[mesh] OR “fungemia”[mesh] OR “viremia”[mesh] OR “acute lung injuries”[tiab] OR “acute lung injury”[tiab] OR “ards”[tiab] OR “artificial respiration”[tiab] OR “artificial ventilation”[tiab] OR “bacteremia”[tiab] OR “bacteremias”[tiab] OR “bacteremic”[tiab] OR “bacteremics”[tiab] OR “blood poisoning”[tiab] OR “blood poisonings”[tiab] OR “burn unit”[tiab] OR “burn units”[tiab] OR “critical care”[tiab] OR “critical illness”[tiab] OR “critical illnesses”[tiab] OR “critically ill”[tiab] OR “endotoxemia”[tiab] OR “endotoxemias”[tiab] OR “endotoxemic”[tiab] OR “endotoxemics”[tiab] OR “fungemia”[tiab] OR “icu”[tiab] OR “intensive care”[tiab] OR “multi organ dysfunction”[tiab] OR “multi organ dysfunctions”[tiab] OR “multi organ failure”[tiab] OR “multi organ failures”[tiab] OR “multi system organ dysfunction”[tiab] OR “multi system organ dysfunctions”[tiab] OR “multi system organ failure”[tiab] OR “multi system organ failures”[tiab] OR “multiorgan dysfunction”[tiab] OR “multiorgan dysfunctions”[tiab] OR “multiorgan failure”[tiab] OR “multiorgan failures”[tiab] OR “multiple organ dysfunction”[tiab] OR “multiple organ dysfunctions”[tiab] OR “multiple organ failure”[tiab] OR “multiple organ failures”[tiab] OR “multisystem organ dysfunction”[tiab] OR “multisystem organ dysfunctions”[tiab] OR “multisystem organ failure”[tiab] OR “multisystem organ failures”[tiab] OR “respiratory care unit”[tiab] OR “respiratory care units”[tiab] OR “respiratory distress syndrome”[tiab] OR “respiratory distress syndromes”[tiab]	840,618

	OR “sepsis”[tiab] OR “septic”[tiab] OR “septicaemia”[tiab] OR “septicaemias”[tiab] OR “septicaemic”[tiab] OR “septicaemics”[tiab] OR “septicemia”[tiab] OR “septicemias”[tiab] OR “shock”[tiab] OR “viremia”[tiab] OR “viremias”[tiab] OR “viremic”[tiab] OR “viremics”[tiab] OR bacillaemi*[tiab] OR bacillemi*[tiab] OR bacteraemi*[tiab] OR fungaemi*[tiab] OR fungemi*[tiab] OR intubat*[tiab] OR mechanical ventilat*[tiab] OR mechanically ventilat*[tiab])	
#2	fentanyl[mesh] OR fentanyl[tiab] OR fenta*[tiab]	26,270
#3	morphine[mesh] OR morphine[tiab] OR morphines[tiab] OR remifentanil[mesh] OR remifentanil[tiab] OR remifentanils[tiab] OR hydromorphone[mesh] OR hydromorphone[tiab] OR hydromorphones[tiab] OR meperidine[mesh] OR meperidine[tiab] OR meperidines[tiab] OR codeine[mesh] OR codeine[tiab] OR codeines[tiab] OR tramadol[mesh] OR tramadol[tiab] OR tramadols[tiab] OR buprenorphine[mesh] OR buprenorphine[tiab] OR buprenorphines[tiab] OR oxycodone[mesh] OR oxycodone[tiab] OR oxycodones[tiab] OR narcotics[mesh] OR narcotics[tiab] OR “analgesics, opioid”[mesh] OR opioid[tiab] OR opioids[tiab] OR “opiate alkaloids”[mesh] OR “opiate alkaloids”[tiab] OR opiate[tiab]	205,344
#4	(randomized controlled trial[pt] OR controlled clinical trial[pt] OR randomized[tiab] OR placebo[tiab] OR drug therapy[sh] OR randomly[tiab] OR trial[tiab] OR groups[tiab] NOT (animals [mh] NOT humans [mh]))	4,446,611
#5	neonate[mesh] OR neonate[tiab] OR neonates[tiab] OR child[mesh] OR child[tiab] OR childs[tiab] OR children[mesh] OR children[tiab] OR infant[mesh] OR infant[tiab] OR infants[tiab]	2,994,027
#6	#1 AND #2 AND #3 AND #4 NOT #5	730

CENTRAL search strategy (search date: 22 June 2021)

#1	MeSH descriptor: [acute lung injury] explode all trees	517
#2	MeSH descriptor: [burn units] explode all trees	45
#3	MeSH descriptor: [critical care] explode all trees	2116
#4	MeSH descriptor: [critical illness] explode all trees	2367
#5	MeSH descriptor: [intensive care units] explode all trees	3749
#6	MeSH descriptor: [intubation] explode all trees	5321

#7	MeSH descriptor: [intubation, intratracheal] explode all trees	4475
#8	MeSH descriptor: [multiple organ failure] explode all trees	418
#9	MeSH descriptor: [respiratory care units] explode all trees	13
#10	MeSH descriptor: [respiration, artificial] explode all trees	6431
#11	MeSH descriptor: [respiratory distress syndrome] explode all trees	2508
#12	MeSH descriptor: [sepsis] explode all trees	4653
#13	MeSH descriptor: [shock] explode all trees	2360
#14	MeSH descriptor: [shock, septic] explode all trees	1000
#15	MeSH descriptor: [ventilators, mechanical] explode all trees	273
#16	MeSH descriptor: [bacteremia] explode all trees	1004
#17	MeSH descriptor: [endotoxemia] in all trees	262
#18	MeSH descriptor: [hemorrhagic septicemia] in all trees	0
#19	MeSH descriptor: [fungemia] explode all trees	88
#20	MeSH descriptor: [viremia] in all trees	364
#21	("acute lung injuries"):ti,ab,kw	3
#22	("acute lung injury"):ti,ab,kw	1280
#23	(ards):ti,ab,kw	2094
#24	("artificial respiration"):ti,ab,kw	49
#25	("artificial ventilation"):ti,ab,kw	5904
#26	(bacteremia):ti,ab,kw	2532
#27	(bacteremias):ti,ab,kw	168
#28	(bacteremic):ti,ab,kw	120
#29	(bacteremics):ti,ab,kw	0
#30	("blood poisoning"):ti,ab,kw	58
#31	("blood poisonings"):ti,ab,kw	0
#32	("burn unit"):ti,ab,kw	89
#33	("burn units"):ti,ab,kw	64
#34	("critical care"):ti,ab,kw	4075
#35	("critical illness"):ti,ab,kw	3674
#36	("critical illnesses"):ti,ab,kw	36
#37	("critically ill"):ti,ab,kw	7174
#38	(endotoxemia):ti,ab,kw	682
#39	(endotoxemias):ti,ab,kw	1
#40	(endotoxemic):ti,ab,kw	24
#41	(endotoxemics):ti,ab,kw	0

#42	(fungemia):ti,ab,kw	147
#43	(icu):ti,ab,kw	13,761
#44	(“intensive care”):ti,ab,kw	24,568
#45	(“multi organ dysfunction”):ti,ab,kw	87
#46	(“multi organ dysfunctions”):ti,ab,kw	0
#47	(“multi organ failure”):ti,ab,kw	272
#48	(“multi organ failures”):ti,ab,kw	2
#49	(“multi system organ failure”):ti,ab,kw	6
#50	(“multi system organ failures”):ti,ab,kw	0
#51	(“multiorgan dysfunction”):ti,ab,kw	144
#52	(“multi organ dysfunctions”):ti,ab,kw	0
#53	(“multiorgan failure”):ti,ab,kw	422
#54	(“multiorgan failures”):ti,ab,kw	2
#55	(“multiple organ dysfunction”):ti,ab,kw	421
#56	(“multiple organ dysfunctions”):ti,ab,kw	10
#57	(“multiple organ failure”):ti,ab,kw	1450
#58	(“multiple organ failures”):ti,ab,kw	8
#59	(“respiratory care unit”):ti,ab,kw	16
#60	(“respiratory care units”):ti,ab,kw	16
#61	(“respiratory distress syndrome”):ti,ab,kw	5558
#62	(“respiratory distress syndromes”):ti,ab,kw	3
#63	(sepsis):ti,ab,kw	11,535
#64	(septic):ti,ab,kw	4714
#65	(shock):ti,ab,kw	11,109
#66	(viremia):ti,ab,kw	1558
#67	(viremias):ti,ab,kw	14
#68	(viremic):ti,ab,kw	202
#69	(viremics):ti,ab,kw	1
#70	#1 OR #2 OR #3 OR #4 OR #5 OR #6 OR #7 OR #8 OR #9 OR #10 OR #11 OR #12 OR #13 OR #14 OR #15 OR #16 OR #17 OR #18 OR #19 OR #20 OR #21 OR #22 OR #23 OR #24 OR #25 OR #26 OR #27 OR #28 OR #29 OR #30 OR #31 OR #32 OR #33 OR #34 OR #35 OR #36 OR #37 OR #38 OR #39 OR #40 OR #41 OR #42 OR #43 OR #44 OR #45 OR #46 OR #47 OR #48 OR #49 OR #50 OR #51 OR #52 OR #53 OR #54 OR #55 OR #56 OR #57 OR #58	69,826

	OR #59 OR #60 OR #61 OR #62 OR #63 OR #64 OR #65 OR #66 OR #67 OR #68 OR #69	
#71	MeSH descriptor: [fentanyl] in all trees	5637
#72	(fentanyl):ti,ab,kw	14,898
#73	#71 OR #72	16,118
#74	MeSH descriptor: [morphine] in all trees	5076
#75	(morphine):ti,ab,kw	14,834
#76	MeSH descriptor: [remifentanil] in all trees	1778
#77	(remifentanil):ti,ab,kw	4816
#78	MeSH descriptor: [hydromorphone] in all trees	398
#79	(hydromorphone):ti,ab,kw	1050
#80	MeSH descriptor: [meperidine] in all trees	1165
#81	(meperidine):ti,ab,kw	1941
#82	MeSH descriptor: [codeine] in all trees	1748
#83	(codeine):ti,ab,kw	1571
#84	MeSH descriptor: [tramadol] in all trees	1170
#85	(tramadol):ti,ab,kw	3963
#86	MeSH descriptor: [buprenorphine] in all trees	1176
#87	(buprenorphine):ti,ab,kw	2594
#88	MeSH descriptor: [oxycodone] in all trees	958
#89	(oxycodone):ti,ab,kw	2506
#90	MeSH descriptor: [narcotics] in all trees	8621
#91	(narcotics):ti,ab,kw	2173
#92	MeSH descriptor: [analgesics, opioid] in all trees	7797
#93	(opioid):ti,ab,kw	22,018
#94	(opioids):ti,ab,kw	7961
#95	MeSH descriptor: [opiate alkaloids] in all trees	11,084
#96	(“opiate alkaloids”):ti,ab,kw	25
#97	(opiate):ti,ab,kw	5952
#98	#74 OR #75 OR #76 OR #77 OR #78 OR #79 OR #80 OR #81 OR #82 OR #83 OR #84 OR #85 OR #86 OR #87 OR #88 OR #89 OR #90 OR #91 OR #92 OR #93 OR #94 OR #95 OR #96 OR #97	46,046
#99	MeSH descriptor: [Infant, Newborn] in all trees	16,362
#100	(neonate):ti,ab,kw	1955
#101	MeSH descriptor: [child] in all trees	57,420

#102	(child):ti,ab,kw	152,756
#103	MeSH descriptor: [children] in all trees	57,420
#104	(children):ti,ab,kw	152,754
#105	MeSH descriptor: [infant] in all trees	32,816
#106	(infant):ti,ab,kw	51,506
#107	#99 OR #100 OR #101 OR #102 OR #103 OR #104 OR #105 OR #106	176,457
#108	#70 AND #73 AND #98 NOT #107	620

Igaku Chuo Zasshi search strategy (search date: 22 June 2021)

#1	((敗血症/TH or 敗血症/AL)) and (PT=会議録除く)	25,018
#2	((ショック/TH or ショック/AL)) and (PT=会議録除く)	35,567
#3	((菌血症/TH or 菌血症/AL)) and (PT=会議録除く)	4581
#4	((人工呼吸/TH or 人工呼吸/TA)) and (PT=会議録除く)	36,583
#5	((人工呼吸器/TH or 人工呼吸器/TA)) and (PT=会議録除く)	15,498
#6	#1 or #2 or #3 or #4 or #5	87,102
#7	((オピオイド系鎮痛剤/TH or オピオイド系鎮痛剤/TA)) and (PT=会議録除く)	17,008
#8	ランダム化比較試験/TH or 準ランダム化比較試験/TH or ランダム化/AL or 無作為化/AL or 比較試験/AL or 臨床試験/AL or プラセボ/AL or 対照/AL or コントロール/AL or 臨床研究/AL	329,496
#9	#7 and #8 and #9	45

Clinical Trials.gov search strategy (search date: 22 June 2021)

Advanced search, no date limit applied.

・Condition or disease: “critical care” OR “intensive care” OR “multiple organ failure” OR “respiratory distress syndrome” OR “sepsis” OR “shock” OR “ventilators, mechanical”.

・Study type: interventional studies.

・Intervention/treatment:

fentanyl OR morphine OR hydromorphone OR meperidine OR codeine OR tramadol OR buprenorphine OR oxycodone OR narcotics OR opioid OR opioids OR opiate.

・Age: adult (18-64 years).

・Status: recruitment and expanded access were checked.

Thirty-two studies were searched.

WHO International Clinical Trials Registry Platform Search strategy (search date: 22 June

2021)

Advanced search

(‘critical care” OR “intensive care” OR “multiple organ failure” OR “respiratory distress syndrome” OR “sepsis” OR “shock” OR “ventilators, mechanical”) in the Condition

AND

(fentanyl OR morphine OR hydromorphone OR meperidine OR codeine OR tramadol OR buprenorphine OR oxycodone OR narcotics OR opioid OR opioids OR opiate) in the Intervention

Six studies were searched.
