# Supplementary material for: Effects of fentanyl administration in mechanically ventilated patients in the intensive care unit: a systematic review and meta-analysis
Source: BMC Anesthesiol. 2022 Oct 21;22:323. doi: 10.1186/s12871-022-01871-7 (PMC9585711; doi:10.1186/s12871-022-01871-7)
Supplement: Supplementary file 2 — Additional file 2. Relevant excluded studies and reasons for exclusion. [file 12871_2022_1871_MOESM2_ESM.pdf]

Additional file 2. Relevant excluded studies and reasons for exclusion.

Study	Journal	Year	PMID	Patients' conditions	Interventions	Comparisons	Reasons for exclusion
Balakrishnan et al.	Anesth Analg	2000	10866905	Adult patients scheduled for nonemergent surgical treatment of an intracranial space-occupying lesion under general anesthesia	Fentanyl	Remifentanil	Interventions were excluded because they were only intraoperative and did not mention ICU management
Breen et al.	Crit Care	2005	15987391	The target population were patients requiring long-term mechanical ventilation for medical reasons	Midazolam-based sedation regime with fentanyl or morphine added for analgesia	Remifentanil-based sedation regime titrated to response before the addition of midazolam for further sedation	Fentanyl and morphine were mixed in the intervention group, but we could not analyze these 2 opioids separately; therefore, this study was excluded
Cheng et al.	Anesth Analg	2001	11323328	ASA physical status III-IV subjects undergoing elective CABG surgery, with cardiopulmonary bypass	A typical fentanyl/isoflurane/propofol regimen	A remifentanil/isoflurane/propofol regimen	This study was excluded because the intervention was only during surgery.

Engoren et al.	Anesth Analg	2001	11574346	Adult patients who underwent cardiac surgery	Fentanyl-based anesthetic	Sufentanil-based or remifentanil-based anesthetic	We excluded this study because the number of patients in each group was not specified, and remifentanil appeared to be continued until the time of ICU admission, but fentanyl and sufentanil were not
Fleisher et al.	J Clin Anesth	2001	11578882	Scheduled for elective surgeries under general endotracheal anesthesia, with an expected duration of unconsciousness of ≥ 30 min	Fentanyl	Remifentanil	This study was excluded because the intervention was only during surgery, and analgesia with morphine or fentanyl was provided in the remifentanil group as transition analgesia
Howie et al.	Anesth Analg	2001	11323327	Patients scheduled for elective CABG surgery with an ASA physical status of III-IV	Fentanyl	Remifentanil	This study was excluded because the intervention was only during surgery, and the same treatment was conducted during the ICU stay
Joshi et al.	J Clin Anesth	2002	12477583	Adult patients scheduled for elective surgical procedures with general anesthesia	Fentanyl treatment	Remifentanil treatment	This study was excluded because the intervention was only during surgery, and the same treatment was conducted during the ICU stay
Kahn et al.	J Burn Care Res	2011	21240000	All burn patients admitted to the burn/trauma ICU	Fentanyl	Morphine	This study was excluded because it was a retrospective study. However, there was no mention of the study design in the title and abstract; Therefore, we did not exclude it until the second screening

Khanykin et al.	Heart Surg Forum	2013	24370801	Elective patients scheduled to undergo CABG, aortic valve replacement, or mitral valve surgery for the first time	Low-dose fentanyl group	Remifentanil	This study was excluded because the intervention was only during surgery.
Maddali et al.	J Clin Anesth	2006	17175431	Primary CABG patients undergoing first-time surgery with cardiopulmonary bypass	Fentanyl	Remifentanil	This study was excluded because the intervention was only during surgery.
Möllhoff et al.	Br J Anaesth	2001	11878522	Elective CABG surgery	Fentanyl	Remifentanil	This study was excluded because the intervention was only during surgery.
Murphy et al.	Anesth Analg	2009	19608797	Patients who underwent cardiac surgery with cardiopulmonary bypass	Fentanyl (600 μg)	Morphine (40 mg)	This study was excluded because the intervention was only during surgery, and the same treatment was conducted during the ICU stay
Myles et al.	Anesth Analg	2002	12351249	Eligible patients for elective CABG	A fentanyl bolus with a small dose of 12 μg/kg (Group FLD), and a fentanyl bolus with a moderate dose of 24 μg/kg (Group FMD)	Remifentanil infusion at 0.83 μg/kg/min (Group R)	This study was excluded because the intervention was only during surgery, and the same treatment was conducted during ICU stay

ICU, intensive care unit; CABG, coronary artery bypass grafting; ASA, American Society of Anesthesiologists.
