# Supplementary material for: Effects of fentanyl administration in mechanically ventilated patients in the intensive care unit: a systematic review and meta-analysis
Source: BMC Anesthesiol. 2022 Oct 21;22:323. doi: 10.1186/s12871-022-01871-7 (PMC9585711; doi:10.1186/s12871-022-01871-7)
Supplement: Supplementary file 3 — Additional file 3. Characteristics of included patients, and details of sedatives and opioids. [file 12871_2022_1871_MOESM3_ESM.pdf]

Additional file 3. Characteristics of included patients, and details of sedatives and opioids.

Authors	Intervention and comparison	Age, years	Male sex	GCS score	APACHE II score	Co-intervention sedatives	Opioid regulation
Cevik et al. [18]	Fentanyl	51.88 (20.77)	10/16 (62.5%)	10.06 (4.74)	11.94 (6.4)	Both groups received midazolam infusion at an initial dose of 0.03 mg/kg/h.	If the Ramsey scale score was ≤ 3, the fentanyl dose was increased by 0.01 μg/kg/min.
	Remifentanil	50.63 (25.24)	7/16 (43.8%)	11 (3.97)	9.56 (3.83)		If the Ramsey scale was ≤ 3, the remifentanil dose was increased by 0.05 μg/kg/min.
Karabinis et al. [19]	Fentanyl	49.6 (16.9)	24/37 (64.9%)	8.8 (2.9)	Not stated	Opioids and sedatives were adjusted to achieve SAS targets of 1 to 3. The sedative medication was propofol for the first 3 days of the study. For patients who still required analgesia and sedation after the third day of the study, propofol was discontinued, and midazolam was administered instead.	The infusion rates of fentanyl were not specified in the protocol, but were used as part of routine clinical practice at each investigation site.
	Remifentanil	46.8 (16.3)	44/84 (52.4%)	8.4 (2.7)	Not stated	If the target scores for the patient's comfort (SAS = 1-3; PI = 1-2) were not achieved with remifentanil alone (infusion rate at 18 μg/kg/h), additional sedation was provided by administering propofol, at the investigator's discretion.	Remifentanil infusion was started at an initial rate of 9 μg/kg/h and was increased in increments of at least 1.5 μg/kg/h at intervals of 5 to 10 min, depending on the clinical requirement or the severity of illness up to 18 μg /kg/h.
Liu et al. [20]	Fentanyl	62 (9.96)	17/35 (48.6%)	Not stated	20.2 (5.04)	Midazolam was administered at a loading dose of 0.05 mg/kg followed by 0.02 ± 0.1 mg/kg/h. The sedative effect was assessed every 4 h using the RASS to ensure that the RASS score was within the range of −1 to −3.	Fentanyl 1 μg/kg/h

	Remifentanil	66.11 (11.94)	21/35 (60%)	Not stated	19.2 (4.19)		Remifentanil 1 μg/kg/h
Muellejans et al. [21]	Fentanyl	58.7 (13.9)	52/75 (69.3%)	Not stated	Not stated	Propofol was administered at an initial bolus dose of up to 0.5 mg/kg only when opioids reached the prescribed dose. Thereafter, propofol was administered as a bolus dose of up to 0.5 mg/kg every hour and a continuous dose of 0.5 mg/kg/h, with the continuous dose increased by 25%. In case of excessive sedation (SAS score: < 4), the sustained dose of propofol was reduced by 25%.	Blinded fentanyl (1 μg/kg bolus + 1.5 μg/kg/h infusion at 6 ml/h) was administered initially, followed by titration of the infusion rate in 1-ml/h increments (fentanyl 0.25 μg/kg/h rate increase).
	Remifentanil	61.5 (13.4)	55/77 (71.4%)	Not stated	Not stated		Blinded opioids (placebo bolus dose + 9 μg/kg/h infusion at 6 ml/h) were initially administered, followed by titration of the infusion volume in 1-ml/h increments (remifentanil 1.5 μg/kg/hr) for optimal sedation (SAS score: 4) with an increase in dose.
Muellejans et al. [22]	Fentanyl	66.5 (7.0)	23/33 (69.7%)	Not stated	Not stated	All patients received an initial bolus dose of midazolam (0.03-0.2 mg/kg). Midazolam infusion was then provided at an initial rate of 0.02 to 0.04 mg/kg/h. If the sedative effect was inadequate, the midazolam infusion rate was increased to a maximum of 0.2 mg/kg/h.	Patients received an initial bolus dose of fentanyl at 1 to 2 μg/kg, followed by an infusion at an initial rate of 1 to 2 μg/kg/h. If the analgesic effect was inadequate, the fentanyl dose was increased to a maximum of 7 μg/kg/h.
	Remifentanil	65 (8.1)	30/39 (76.9%)	Not stated	Not stated	If an adequate level of sedation was not achieved with remifentanil alone at an infusion rate of 60 μg/kg/h, additional sedation was provided by administering a bolus dose of propofol (0.3-1.0 mg/kg) and/or propofol infusion starting at a rate of 0.5 to 1.0 mg/kg/h (maximum dose of 4 mg/kg/h).	Remifentanil infusion was continued or started at an initial rate of 6 to 12 μg/kg/h and increased depending on clinical requirements up to 60 μg/kg/h.

Oliver et al. [23]	Fentanyl	62 (55-71)*	25/38 (65.8%)	Not stated	Not stated	Propofol infusion began at 25 g/kg/min, and a standard anesthetic consisting of midazolam 0.1 mg/kg, muscle relaxant, oxygen, and isoflurane in the air was administered.	Intraoperatively, fentanyl was infused at 0.5 µg/kg/h to initiate analgesia. Postoperatively, a combined continuous and bolus fentanyl administration algorithm was used to maintain the VAS score < 3.
	Morphine	63 (53-72)*	25/41 (61.0%)	Not stated	Not stated		Intraoperatively, morphine was administered as a bolus. Postoperatively, the algorithm was administration of a bolus dose of 2 mg morphine to achieve a VAS score of ≤ 3.
Spies et al. [24]	Fentanyl	63 (12)	27/32 (84.4%)	Not stated	26 (9)	A standard protocol was adopted for sedation with a target of a RASS score of 0 to −1. Propofol (up to 4 mg/kg ideal body weight/h) was used for sedation, and midazolam (0.01-0.18 mg/kg ideal body weight/h) was added as required.	Fentanyl was administered at 0.02 to 0.08 µg/kg ideal body weight/min, and a protocol was adopted to adjust the dose by assessing the VAS and the BPS.
	Remifentanil	64 (15)	20/28 (71.4%)	Not stated	24 (8)		Remifentanil was administered at 0.1 to 0.4 µg/kg ideal body weight/min, and a protocol was adopted to adjust the dose by assessing the VAS and the BPS.

GCS, Glasgow Coma Scale; APACHE II, Acute Physiology and Chronic Health Evaluation II; SAS, Sedation–Agitation Scale; PI, pain intensity; MV, mechanical ventilation; RASS, Richmond Agitation Sedation Scale; VAS, Visual Analog Scale; BPS, Behavioral Pain Scale.
Values are number (%) or mean (standard deviation).
*Values are median (interquartile range).
