# Supplementary material for: Effects of fentanyl administration in mechanically ventilated patients in the intensive care unit: a systematic review and meta-analysis
Source: BMC Anesthesiol. 2022 Oct 21;22:323. doi: 10.1186/s12871-022-01871-7 (PMC9585711; doi:10.1186/s12871-022-01871-7)
Supplement: Supplementary file 4 — Additional file 4. Outcomes of included studies. [file 12871_2022_1871_MOESM4_ESM.pdf]

Additional file 4. Outcomes of included studies.

Authors	Intervention and comparison	Mortality, n (%)	Duration of MV (h or min)	Duration of the ICU stay (h or days)	Severe adverse events, n (%)	Delirium, n (%)
Cevik et al. [18]	Fentanyl	Not reported	45.75 (47.13) h	237 (159.89) h	5/16 (31.3)	Not reported
	Remifentanil	Not reported	45.75 (74.71) h	208.69 (239.06) h	7/16 (43.8)	Not reported
Karabinis et al. [19]	Fentanyl	0/37 (0)	24.08 (16.4-70)* h	42.9 (3.7-144.2)* h	2/37 (5.4)	Not reported
	Remifentanil	4/84 (4.8)	24.83 (12.5-110.2)* h	43.5 (2.4-150)* h	4/84 (4.8)	Not reported
Liu et al. [20]	Fentanyl	4/35 (11.4) at 28 days	126 (68-256)** h	7 (5-13)** days	Not reported	14/35 (40)
	Remifentanil	4/35 (11.4) at 28 days	102 (68-157)** h	6 (4-9)** days	Not reported	8/35 (22.9)
Muellejans et al. [21]	Fentanyl	Not reported	1.3 (0.9) h	39.5 (40.5) h	0/81	Not reported

	Remifentanil	Not reported	1.1 (2.8) h	40.8 (40.2) h	0/115	Not reported
Muellejans et al. [22]	Fentanyl	Not reported	24.2 (7.0) h	62.4 (27.2 h)	6/39 (15.4)	9/39 (23.1)
	Remifentanil	Not reported	20.7 (5.2) h	46.1 (22.0) h	4/40 (10)	7/40 (17.5)
Oliver et al. [23]	Fentanyl	Not reported	280 (240-360)** min	23.1 (22.1-24.0)** h	Not reported	Not reported
	Morphine	Not reported	284 (215-345)** min	23.2 (21.6-24.8)** h	Not reported	Not reported
Spies et al. [24]	Fentanyl	Not reported	162 (96-228)*** h	26 (17-35)*** days	Not reported	7 (21.9)
	Remifentanil	Not reported	136 (79-192)*** h	23 (14-32)*** days	Not reported	8 (28.6)

MV, mechanical ventilation; ICU, intensive care unit.
Values are number (%) or mean (standard deviation).
*Values are median (range).
**Values are median (interquartile range).
***Values are mean (95% confidence interval).
†This outcome was calculated as the sum of myocardial infarction, cardiac arrest, and complete heart block.
