# Supplementary material for: Effects of fentanyl administration in mechanically ventilated patients in the intensive care unit: a systematic review and meta-analysis
Source: BMC Anesthesiol. 2022 Oct 21;22:323. doi: 10.1186/s12871-022-01871-7 (PMC9585711; doi:10.1186/s12871-022-01871-7)

Additional file 5-a. Forest plot of all outcomes

Study or Subgroup	Fentanyl Events	Fentanyl Total	Other Opioids Events	Other Opioids Total	Weight	Risk Ratio M-H, Random, 95% CI
**1.1.1 Remifentanil**						
Karabinis et al. (ref. 19)	0	37	4	84	16.9%	0.25 [0.01, 4.50]
Liu et al. (ref. 20)	4	35	4	35	83.1%	1.00 [0.27, 3.69]
**Subtotal (95% CI)**		**72**		**119**	**100.0%**	**0.79 [0.24, 2.60]**
Total events	4		8			
Heterogeneity: Tau² = 0.00; Chi² = 0.79, df = 1 (P = 0.38); I² = 0%						
Test for overall effect: Z = 0.39 (P = 0.70)						
**Total (95% CI)**		**72**		**119**	**100.0%**	**0.79 [0.24, 2.60]**
Total events	4		8			
Heterogeneity: Tau² = 0.00; Chi² = 0.79, df = 1 (P = 0.38); I² = 0%						
Test for overall effect: Z = 0.39 (P = 0.70)						
Test for subgroup differences: Not applicable						

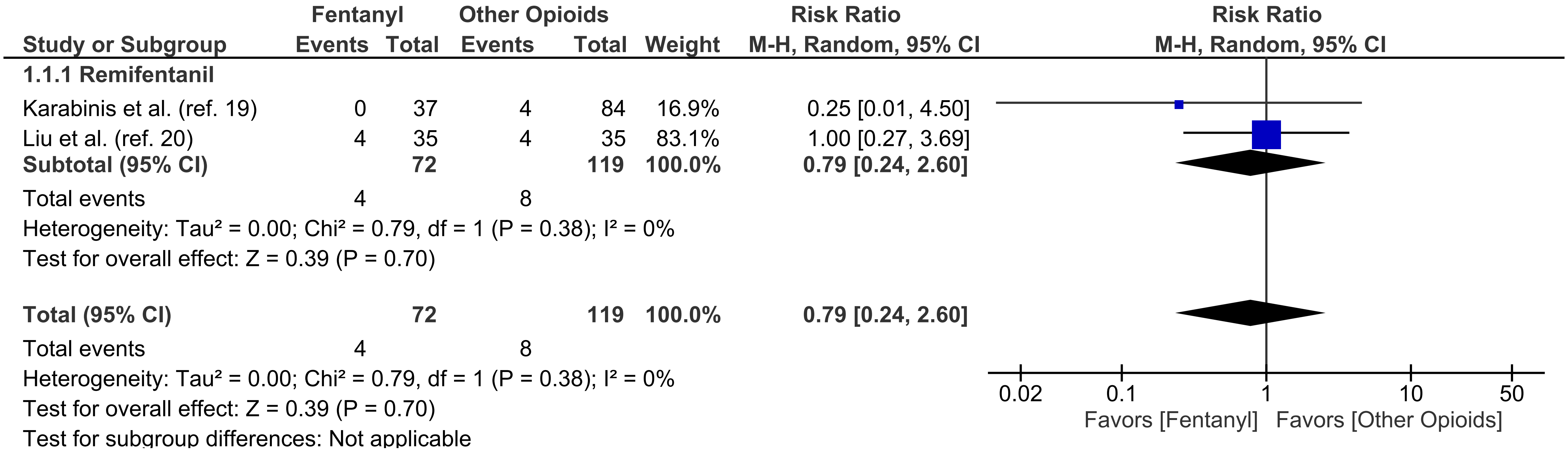

Additional file 5-b. Forest plot of mechanical ventilation

Study or Subgroup	Fentanyl Mean	Fentanyl SD	Fentanyl Total	Other Opioids Mean	Other Opioids SD	Other Opioids Total	Weight	Mean Difference IV, Random, 95% CI
**1.2.1 Remifentanil**								
Cevik et al. (ref. 18)	45.75	47.13	16	45.75	74.71	16	0.1%	0.00 [-43.28, 43.28]
Karabinis et al. (ref. 19)	33.64	14.03	22	43.09	22.01	47	2.5%	-9.45 [-18.05, -0.85]
Liu et al. (ref. 20)	150	145.32	35	109	68.8	35	0.1%	41.00 [-12.27, 94.27]
Muellejans et al. (ref. 21)	1.3	0.9	75	1.1	2.8	77	41.7%	0.20 [-0.46, 0.86]
Muellejans et al. (ref. 22)	24.2	7	33	20.7	5.2	39	15.3%	3.50 [0.61, 6.39]
Spies et al. (ref. 24)	162	66	32	136	56.5	28	0.2%	26.00 [-5.00, 57.00]
**Subtotal (95% CI)**			**213**			**242**	**59.8%**	**0.63 [-3.48, 4.74]**
Heterogeneity: Tau² = 9.75; Chi² = 14.60, df = 5 (P = 0.01); I² = 66%								
Test for overall effect: Z = 0.30 (P = 0.76)								
**1.2.2 Morphine**								
Oliver et al. (ref. 23)	4.75	1.86	38	4.69	1.66	41	40.2%	0.06 [-0.72, 0.84]
**Subtotal (95% CI)**			**38**			**41**	**40.2%**	**0.06 [-0.72, 0.84]**
Heterogeneity: Not applicable								
Test for overall effect: Z = 0.15 (P = 0.88)								
**Total (95% CI)**			**251**			**283**	**100.0%**	**0.49 [-0.90, 1.88]**
Heterogeneity: Tau² = 1.09; Chi² = 14.87, df = 6 (P = 0.02); I² = 60%								
Test for overall effect: Z = 0.69 (P = 0.49)								
Test for subgroup differences: Chi² = 0.07, df = 1 (P = 0.79), I² = 0%								

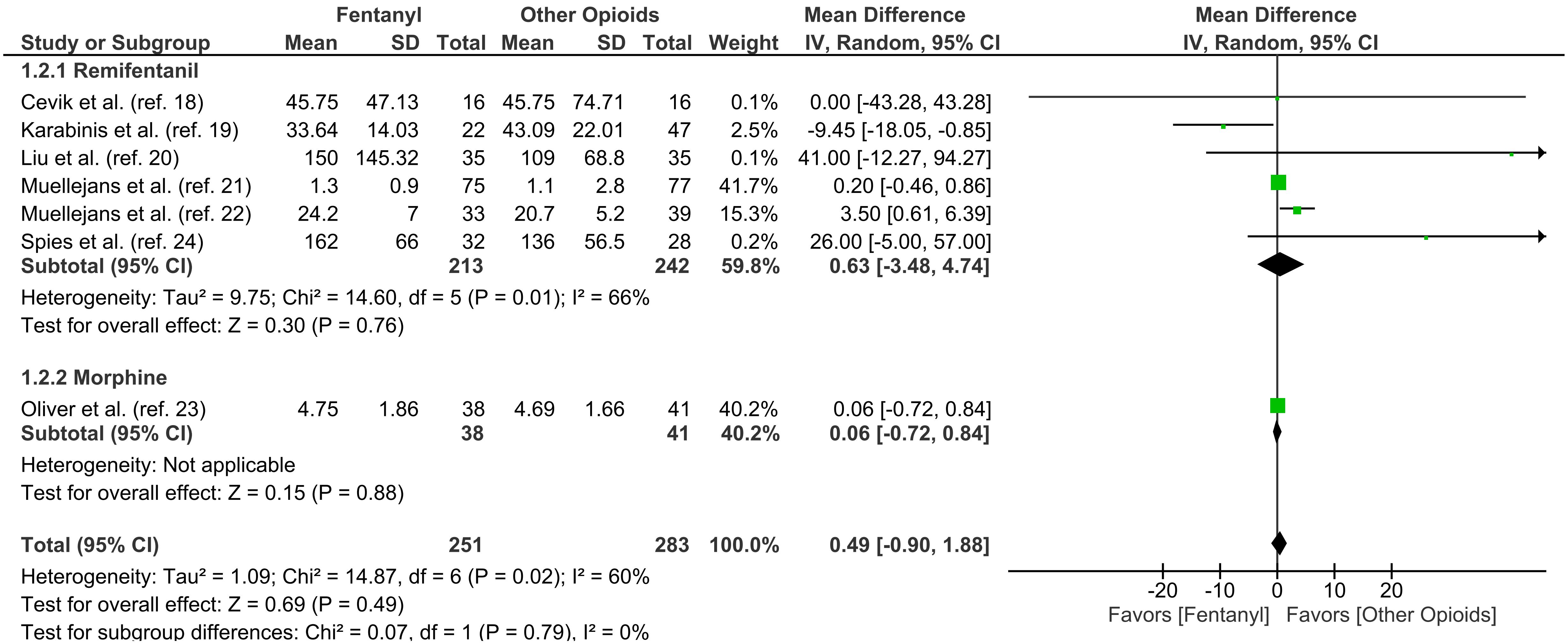

Additional file 5-c. Forest plot of duration of the ICU stay

Study or Subgroup	Fentanyl Mean	Fentanyl SD	Fentanyl Total	Other Opioids Mean	Other Opioids SD	Other Opioids Total	Weight	Mean Difference IV, Random, 95% CI
**1.3.1 Remifentanil**								
Cevik et al. (ref. 18)	237	159.89	16	208.69	239.06	16	0.5%	28.31 [-112.61, 169.23]
Karabinis et al. (ref. 19)	58.43	36.79	22	59.85	33.25	47	16.3%	-1.42 [-19.49, 16.65]
Liu et al. (ref. 20)	200	148.41	35	152	92.76	35	2.9%	48.00 [-9.98, 105.98]
Muellejans et al. (ref. 21)	39.5	40.5	75	40.8	40.2	77	21.6%	-1.30 [-14.13, 11.53]
Muellejans et al. (ref. 22)	62.4	27.2	33	46.1	22	39	23.1%	16.30 [4.73, 27.87]
Spies et al. (ref. 24)	624	108	32	552	108	28	3.2%	72.00 [17.22, 126.78]
**Subtotal (95% CI)**			**213**			**242**	**67.6%**	**12.29 [-3.16, 27.74]**
Heterogeneity: Tau² = 164.01; Chi² = 12.16, df = 5 (P = 0.03); I² = 59%								
Test for overall effect: Z = 1.56 (P = 0.12)								
**1.3.2 Morphine**								
Oliver et al. (ref. 23)	23.07	1.46	38	23.2	2.46	41	32.4%	-0.13 [-1.01, 0.75]
**Subtotal (95% CI)**			**38**			**41**	**32.4%**	**-0.13 [-1.01, 0.75]**
Heterogeneity: Not applicable								
Test for overall effect: Z = 0.29 (P = 0.77)								
**Total (95% CI)**			**251**			**283**	**100.0%**	**7.04 [-3.27, 17.35]**
Heterogeneity: Tau² = 85.06; Chi² = 17.20, df = 6 (P = 0.009); I² = 65%								
Test for overall effect: Z = 1.34 (P = 0.18)								
Test for subgroup differences: Chi² = 2.47, df = 1 (P = 0.12), I² = 59.6%								

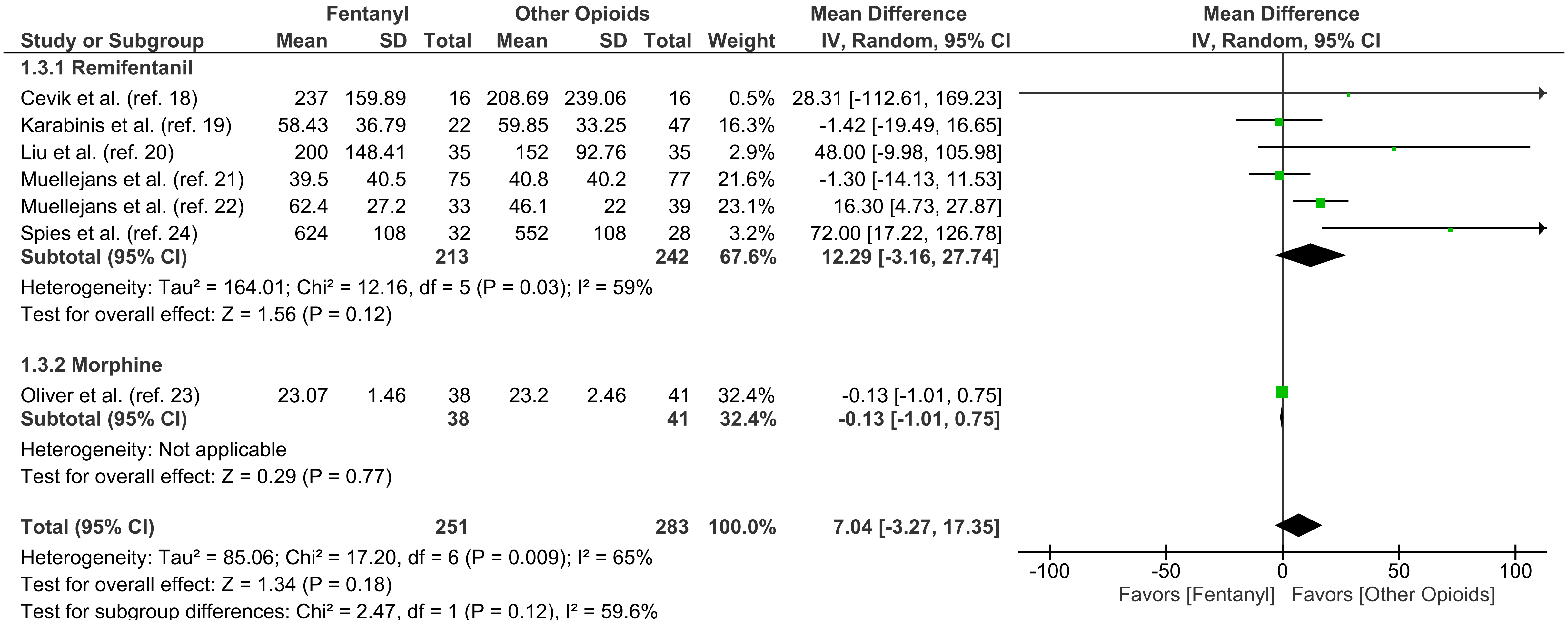

Additional file 5-d. Forest plot of severe adverse events

Study or Subgroup	Fentanyl Events	Fentanyl Total	Other opioids Events	Other opioids Total	Weight	Risk Ratio M-H, Random, 95% CI
**1.4.1 Remifentanil**						
Cevik et al. (ref. 18)	5	16	7	16	52.6%	0.71 [0.29, 1.78]
Karabinis et al. (ref. 19)	2	37	4	84	16.1%	1.14 [0.22, 5.93]
Muellejans et al. (ref. 21)	0	81	0	115		Not estimable
Muellejans et al. (ref. 22)	6	39	4	40	31.3%	1.54 [0.47, 5.04]
**Subtotal (95% CI)**		**173**		**255**	**100.0%**	**0.98 [0.50, 1.90]**
Total events	13		15			

Heterogeneity: Tau² = 0.00; Chi² = 1.07, df = 2 (P = 0.59); I² = 0%
Test for overall effect: Z = 0.06 (P = 0.95)

Study or Subgroup	Fentanyl Events	Fentanyl Total	Other opioids Events	Other opioids Total	Weight	Risk Ratio M-H, Random, 95% CI
**Total (95% CI)**		**173**		**255**	**100.0%**	**0.98 [0.50, 1.90]**
Total events	13		15			

Heterogeneity: Tau² = 0.00; Chi² = 1.07, df = 2 (P = 0.59); I² = 0%
Test for overall effect: Z = 0.06 (P = 0.95)
Test for subgroup differences: Not applicable

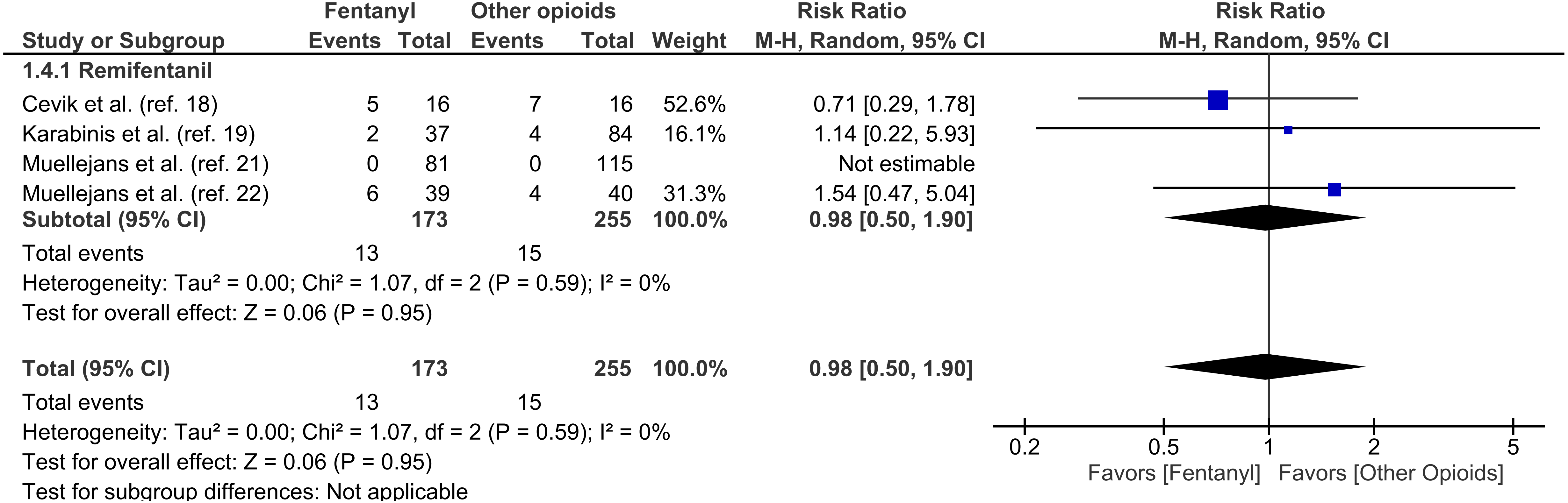

Additional file 5-e. Forest plot of delirium

	Fentanyl		Other Opioids			Risk Ratio
**Study or Subgroup**	**Events**	**Total**	**Events**	**Total**	**Weight**	**M-H, Random, 95% CI**
**1.5.1 Remifentanil**						
Liu et al. (ref. 20)	14	35	8	35	42.0%	1.75 [0.84, 3.64]
Muellejans et al. (ref. 22)	9	39	7	40	28.9%	1.32 [0.54, 3.19]
Spies et al. (ref. 24)	7	32	8	28	29.2%	0.77 [0.32, 1.84]
**Subtotal (95% CI)**		**106**		**103**	**100.0%**	**1.27 [0.79, 2.04]**
Total events	30		23			
Heterogeneity: Tau² = 0.00; Chi² = 2.02, df = 2 (P = 0.36); I² = 1%						
Test for overall effect: Z = 0.97 (P = 0.33)						
**Total (95% CI)**		**106**		**103**	**100.0%**	**1.27 [0.79, 2.04]**
Total events	30		23			
Heterogeneity: Tau² = 0.00; Chi² = 2.02, df = 2 (P = 0.36); I² = 1%						
Test for overall effect: Z = 0.97 (P = 0.33)						
Test for subgroup differences: Not applicable						

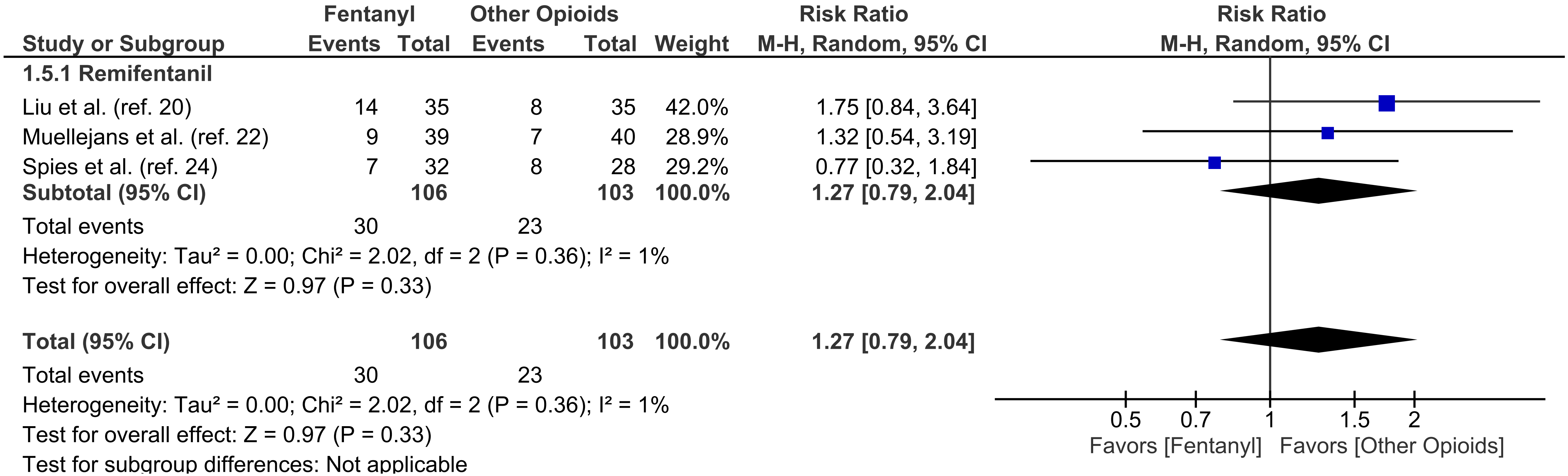

Supplement: Supplementary file 5 — Additional file 5. Forest plot of all outcomes. [file 12871_2022_1871_MOESM5_ESM.pdf]
