# Supplementary material for: Effects of fentanyl administration in mechanically ventilated patients in the intensive care unit: a systematic review and meta-analysis
Source: BMC Anesthesiol. 2022 Oct 21;22:323. doi: 10.1186/s12871-022-01871-7 (PMC9585711; doi:10.1186/s12871-022-01871-7)

Additional file 6-a. Forest plot of all outcomes in the sensitivity analysis

Study or Subgroup	Fentanyl Events	Fentanyl Total	Other Opioids Events	Other Opioids Total	Weight	Risk Ratio M-H, Random, 95% CI
**1.1.1 Remifentanil**						
Liu et al. (ref. 20)	4	35	4	35	100.0%	1.00 [0.27, 3.69]
**Subtotal (95% CI)**		**35**		**35**	**100.0%**	**1.00 [0.27, 3.69]**
Total events	4		4			
Heterogeneity: Not applicable						
Test for overall effect: Z = 0.00 (P = 1.00)						
**Total (95% CI)**		**35**		**35**	**100.0%**	**1.00 [0.27, 3.69]**
Total events	4		4			
Heterogeneity: Not applicable						
Test for overall effect: Z = 0.00 (P = 1.00)						
Test for subgroup differences: Not applicable						

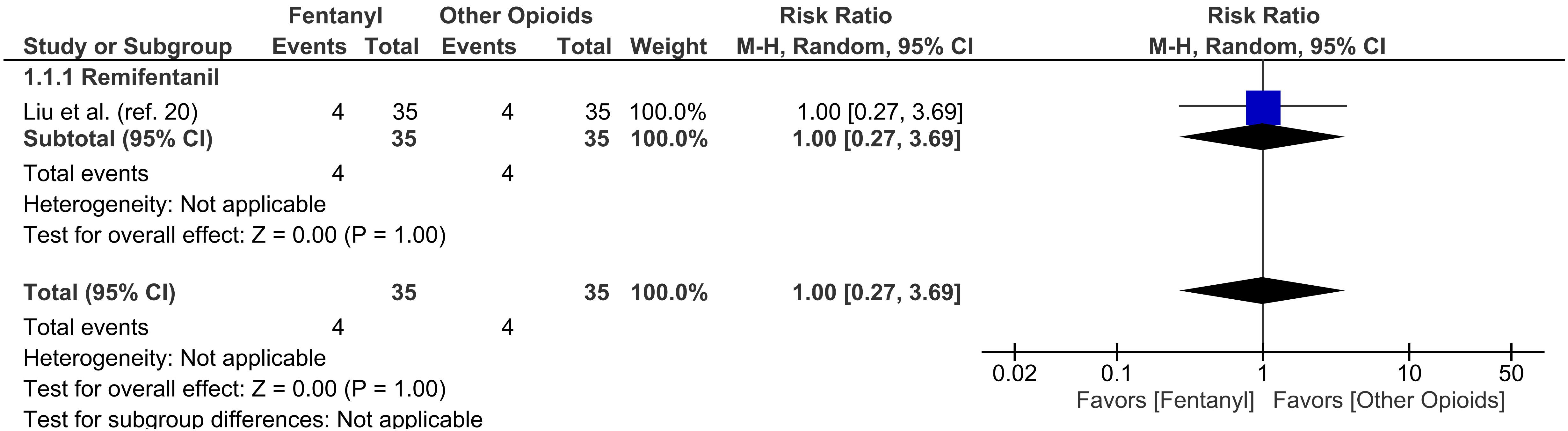

Additional file 6-b. Forest plot of duration of mechanical ventilation excluded different co-interventions

	Fentanyl			Other Opioids				Mean Difference
**Study or Subgroup**	**Mean**	**SD**	**Total**	**Mean**	**SD**	**Total**	**Weight**	**IV, Random, 95% CI**
**1.2.1 Remifentanil**								
Cevik et al. (ref. 18)	45.75	47.13	16	45.75	74.71	16	0.0%	0.00 [-43.28, 43.28]
Liu et al. (ref. 20)	150	145.32	35	109	68.8	35	0.0%	41.00 [-12.27, 94.27]
Muellejans et al. (ref. 21)	1.3	0.9	75	1.1	2.8	77	54.2%	0.20 [-0.46, 0.86]
Spies et al. (ref. 24)	162	66	32	136	56.5	28	0.1%	26.00 [-5.00, 57.00]
**Subtotal (95% CI)**			**158**			**156**	**54.3%**	**8.93 [-8.17, 26.03]**
Heterogeneity: Tau² = 129.24; Chi² = 4.91, df = 3 (P = 0.18); I² = 39%								
Test for overall effect: Z = 1.02 (P = 0.31)								
**1.2.2 Morphine**								
Oliver et al. (ref. 23)	4.75	1.86	38	4.69	1.66	41	45.7%	0.06 [-0.72, 0.84]
**Subtotal (95% CI)**			**38**			**41**	**45.7%**	**0.06 [-0.72, 0.84]**
Heterogeneity: Not applicable								
Test for overall effect: Z = 0.15 (P = 0.88)								
**Total (95% CI)**			**196**			**197**	**100.0%**	**0.16 [-0.56, 0.88]**
Heterogeneity: Tau² = 0.14; Chi² = 5.00, df = 4 (P = 0.29); I² = 20%								
Test for overall effect: Z = 0.43 (P = 0.67)								
Test for subgroup differences: Chi² = 1.03, df = 1 (P = 0.31), I² = 3.0%								

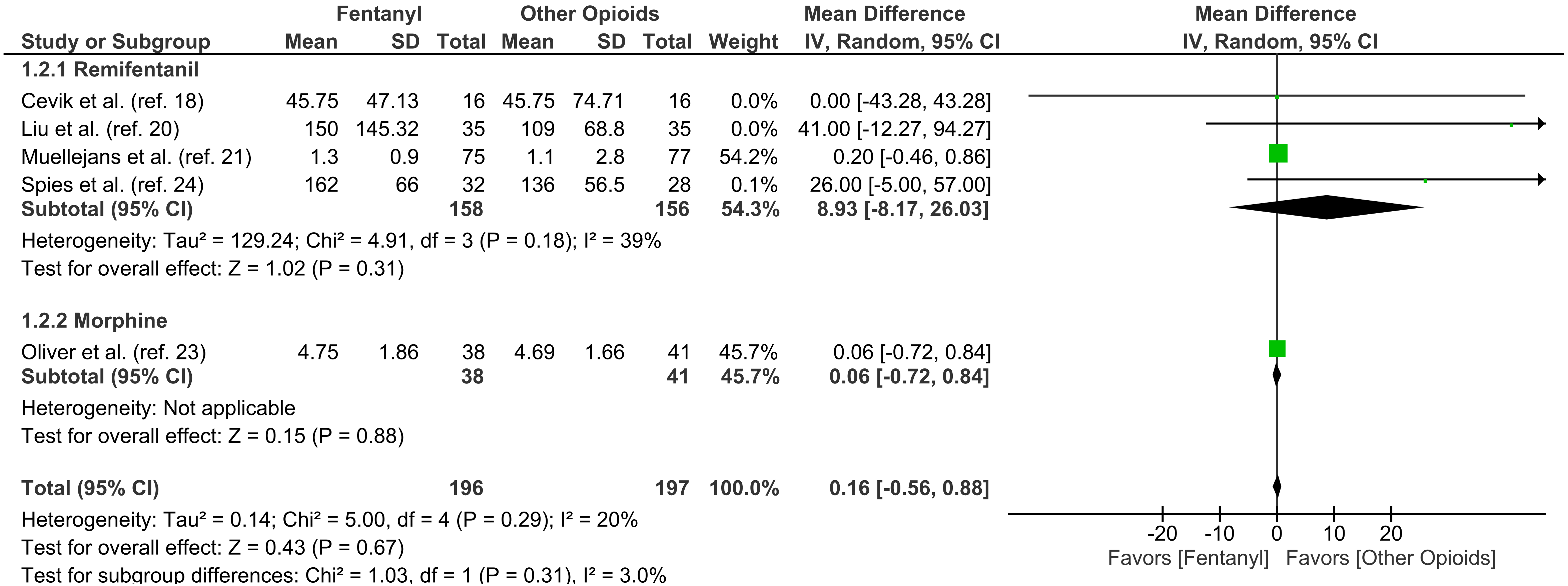

Additional file 6-c. Forest plot of duration of ICU stay excluded different co-interventions

	Fentanyl			Other Opioids				Mean Difference
**Study or Subgroup**	**Mean**	**SD**	**Total**	**Mean**	**SD**	**Total**	**Weight**	**IV, Random, 95% CI**
**1.3.1 Remifentanil**								
Cevik et al. (ref. 18)	237	159.89	16	208.69	239.06	16	0.5%	28.31 [-112.61, 169.23]
Karabinis et al. (ref. 19)	58.43	36.79	22	59.85	33.25	47	16.3%	-1.42 [-19.49, 16.65]
Liu et al. (ref. 20)	200	148.41	35	152	92.76	35	2.9%	48.00 [-9.98, 105.98]
Muellejans et al. (ref. 21)	39.5	40.5	75	40.8	40.2	77	21.6%	-1.30 [-14.13, 11.53]
Muellejans et al. (ref. 22)	62.4	27.2	33	46.1	22	39	23.1%	16.30 [4.73, 27.87]
Spies et al. (ref. 24)	624	108	32	552	108	28	3.2%	72.00 [17.22, 126.78]
**Subtotal (95% CI)**			**213**			**242**	**67.6%**	**12.29 [-3.16, 27.74]**
Heterogeneity: $Tau^2 = 164.01$; $Chi^2 = 12.16$, df = 5 (P = 0.03); $I^2 = 59\%$								
Test for overall effect: Z = 1.56 (P = 0.12)								
**1.3.2 Morphine**								
Oliver et al. (ref. 23)	23.07	1.46	38	23.2	2.46	41	32.4%	-0.13 [-1.01, 0.75]
**Subtotal (95% CI)**			**38**			**41**	**32.4%**	**-0.13 [-1.01, 0.75]**
Heterogeneity: Not applicable								
Test for overall effect: Z = 0.29 (P = 0.77)								
**Total (95% CI)**			**251**			**283**	**100.0%**	**7.04 [-3.27, 17.35]**
Heterogeneity: $Tau^2 = 85.06$; $Chi^2 = 17.20$, df = 6 (P = 0.009); $I^2 = 65\%$								
Test for overall effect: Z = 1.34 (P = 0.18)								
Test for subgroup differences: $Chi^2 = 2.47$, df = 1 (P = 0.12), $I^2 = 59.6\%$								

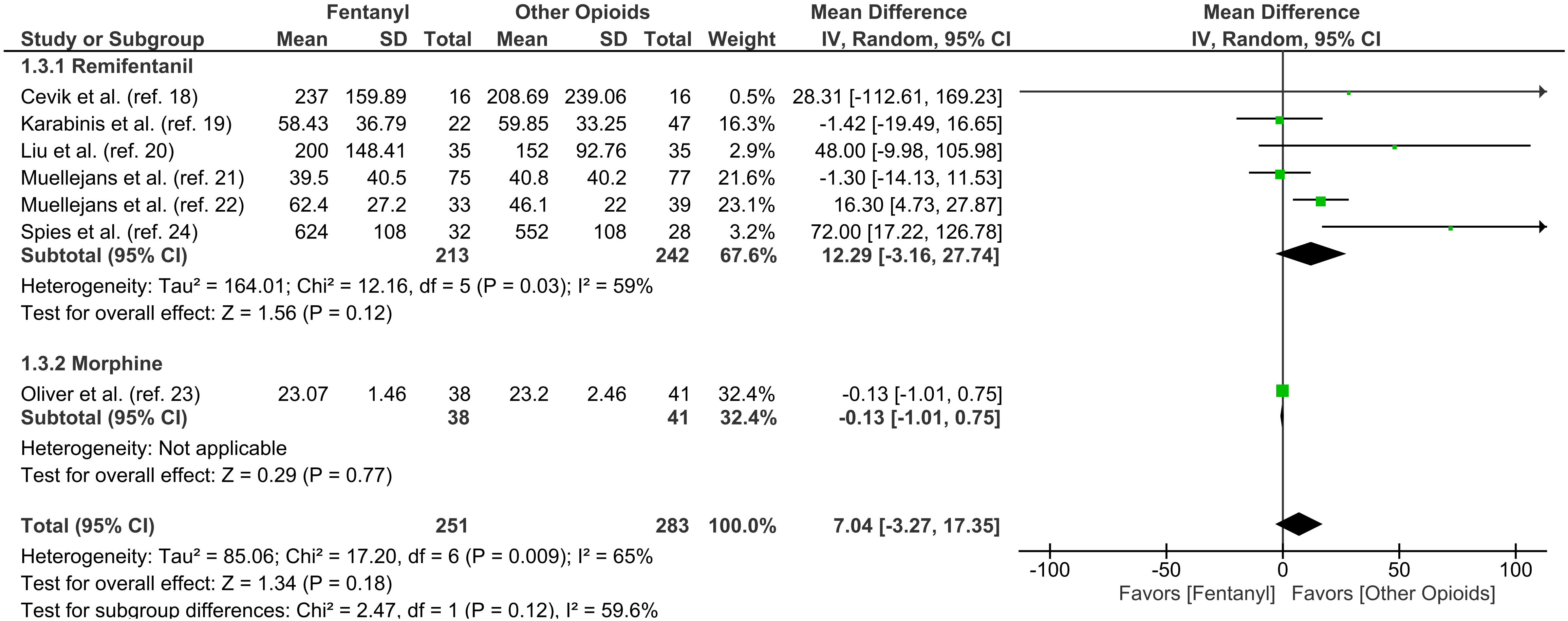

Additional file 6-d. Forest plot of severe adverse events excluded different co-interventions

Study or Subgroup	Fentanyl Events	Fentanyl Total	Other opioids Events	Other opioids Total	Weight	Risk Ratio M-H, Random, 95% CI
**1.4.1 Remifentanil**						
Cevik et al. (ref. 18)	5	16	7	16	100.0%	0.71 [0.29, 1.78]
Muellejans et al. (ref. 21)	0	81	0	115		Not estimable
**Subtotal (95% CI)**		**97**		**131**	**100.0%**	**0.71 [0.29, 1.78]**
Total events	5		7			
Heterogeneity: Not applicable						
Test for overall effect: Z = 0.72 (P = 0.47)						
**Total (95% CI)**		**97**		**131**	**100.0%**	**0.71 [0.29, 1.78]**
Total events	5		7			
Heterogeneity: Not applicable						
Test for overall effect: Z = 0.72 (P = 0.47)						
Test for subgroup differences: Not applicable						

Risk Ratio
M-H, Random, 95% CI

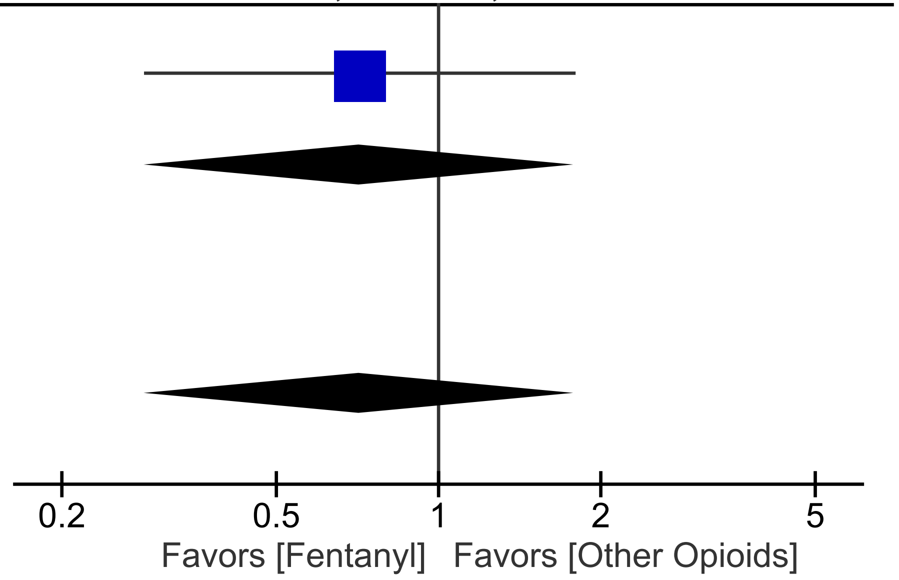

Additional file 6-e. Forest plot of delirium excluded different co-interventions

Study or Subgroup	Fentanyl Events	Fentanyl Total	Other Opioids Events	Other Opioids Total	Weight	Risk Ratio M-H, Random, 95% CI
**1.5.1 Remifentanil**						
Liu et al. (ref. 20)	14	35	8	35	54.5%	1.75 [0.84, 3.64]
Spies et al. (ref. 24)	7	32	8	28	45.5%	0.77 [0.32, 1.84]
**Subtotal (95% CI)**		**67**		**63**	**100.0%**	**1.20 [0.54, 2.69]**
Total events	21		16			
Heterogeneity: Tau² = 0.17; Chi² = 2.01, df = 1 (P = 0.16); I² = 50%						
Test for overall effect: Z = 0.45 (P = 0.66)						
**Total (95% CI)**		**67**		**63**	**100.0%**	**1.20 [0.54, 2.69]**
Total events	21		16			
Heterogeneity: Tau² = 0.17; Chi² = 2.01, df = 1 (P = 0.16); I² = 50%						
Test for overall effect: Z = 0.45 (P = 0.66)						
Test for subgroup differences: Not applicable						

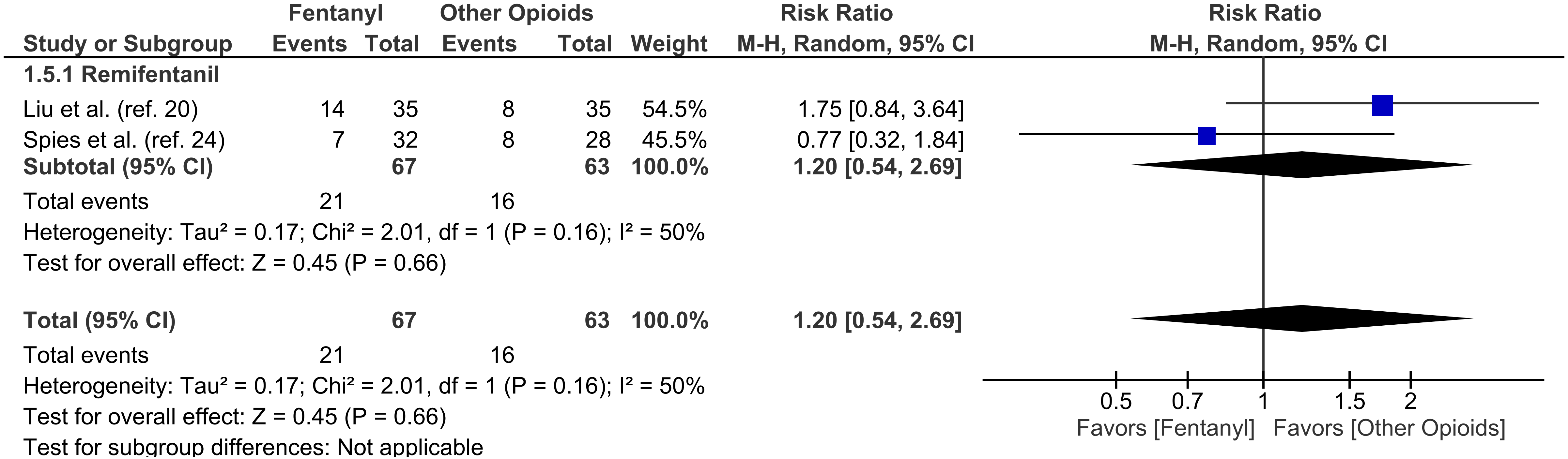

Supplement: Supplementary file 6 — Additional file 6. Forest plot of all outcomes in the sensitivity analysis. [file 12871_2022_1871_MOESM6_ESM.pdf]
